# The Carotenoid Compound of Saffron Crocetin Alleviates Effects of Ischemia Reperfusion Injury via a Mechanism Possibly Involving MiR-127

**DOI:** 10.7759/cureus.6979

**Published:** 2020-02-13

**Authors:** Constantinos P Michael, Michael Derpapas, Eftychia Aravidou, Michael Sofopoulos, Panayiotis Michael, Andreas Polydorou, Antonios Vezakis, Georgios P Fragulidis

**Affiliations:** 1 Medicine, National and Kapodistrian University of Athens School of Medicine, Athens, GRC; 2 Breast Surgery, REA Maternity Hospital, Athens, GRC; 3 Surgery, Aretaeio Hospital, National and Kapodistrian University of Athens School of Medicine, Athens, GRC; 4 Pathology, "Saint Savvas" Regional Anticancer Hospital, Athens, GRC; 5 Mathematics, Cyprus Ministry of Education and Culture, Nicosia, CYP

**Keywords:** mir-127, saffron, crocetin, ischemia, reperfusion, injury, renal failure, micrornas, pcr, ischemic shock

## Abstract

Renal impairment is associated with high mortality rates in severely ill patients. The need to prevent and treat renal damage underlines the importance of understanding the pathophysiological mechanisms that characterize it. This could also enable early diagnosis and the design of alternative therapeutic approaches. The aim of this study is to investigate the effect of crocetin, a known antioxidant, on the prevention of renal damage due to ischemia-reperfusion injury and the investigation of the mechanisms involved. The present study was performed on C57BL/6 mice aged 10-12 weeks. The animals had access to water and food ad libitum. The experiment, as described in materials and methods, was completed at 24 h, in which case the kidneys were removed for further study, both at tissue morphology (with immunohistochemistry) and changes in the level of miRs’ expression by qRT-PCR. Accordingly, using the automatic precision analyzer, the serum levels of the basic parameters currently used clinically for the monitoring of renal function were determined. The administration of crocetin, despite the short presence of the substance in the body, affects all the biochemical parameters analyzed (urea, creatinine, uric acid, and ions of Na, K, Cl, P, Mg and Ca), ​​causing significant decrease of their measured values. Crocetin also resulted in a significant limitation of the inflammation elements and the degree of epithelial damage. Furthermore, the administration of crocetin appears to restore levels of expression of miR21, miR127 and miR132.

## Introduction

Ischemia reperfusion injury (IRI) is one of the main mechanisms for causing acute renal failure in preclinical studies [1]. A number of clinical entities that causes renal hypoxia have a frequent incidence of renal parenchymal lesions and limiting renal function. Renal hypoxia is involved in clinical conditions that either reduce blood supply to the kidney such as hypovolemia (massive bleeding, dehydration) and shock (septic, cardiovascular) or complete discontinuation of renal perfusion such as a series of surgical procedures requiring complete cessation of blood circulation in the renal arteries (kidney transplantation, partial nephrectomy, abdominal aortic aneurysms above the renal artery excretion, etc.) [2-4].

Renal ischemia causes simultaneous lesions in renal parenchyma and renal vessels and triggers an acute inflammatory reaction which in turn will cause further cellular damage. During reperfusion, the tissue continues to undergo lesions other than those caused by the preceding ischemia. As a result, renal cells are lost through apoptosis and cell necrosis [5]. The degree of renal damage caused will predetermine the final degree of renal function, which may eventually reach up to full kidney failure.

Three major parallel pathophysiological mechanisms have been implicated [6,7]. The first is related to the reaction of the vascular limb of the kidney to ischemia. Prolonged vasoconstriction and disruption of normal endothelial cell function is implicated in this segment. The second mechanism has to do with agglomerates of damaged cylindrical epithelial cells that block the renal tubules and cause retrograde flow of the permeate from the tubes back to blood circulation. The third refers to the damage caused by the return of blood flow and oxygen to the renal parenchyma during reperfusion. Reperfusion is necessary for the survival of renal tissue, but is believed to be directly linked to additional lesions due to free oxygen radicals produced in the kidney.

Crocetin, an important carotenoid constituent of saffron, has been reported to have multiple medical properties as it increases oxygen diffusion, recovers cellular adenosine triphosphate (ATP) and scavenging oxygen free radicals [8-13]. The study utilized biochemical parameters, histological parameters and selected microRNAs. The aim of this study is to investigate the effect of crocetin, a known antioxidant, on the prevention of renal damage due to ischemia-reperfusion and the investigation of the mechanisms involved.

## Materials and methods

Animals and IRI model

All animal procedures were performed in accordance to the guidelines of the Ethical Committee of Aretaeio Hospital (Licence Number 5486/23-12-2011 and Ethical Committee Licence M-52/26-05-2011). Mice were kept on 12-h light-dark cycles with free access to food and water ad libitum. Male, B57/BL6 mice, 10-12 week old, were randomly divided into four groups (n = 5 each): sham group (surgical preparation, no shock, and no resuscitation), sham group with crocetin administration, control group (ischemia and resuscitation), and crocetin group (ischemia /resuscitation and treated with crocetin). On the day of experimentation, mice were put to anesthesia by intraperitoneal injection of Ketamine (90 mg/kg bodyweight) and Xylazine (7.5 mg/kg bodyweight). After anesthesia, the right kidneys were surgically revealed. Using renal pedicle clamps to block blood stream from renal artery, ischemic shock was induced. After 45 min, having phenotypically confirmed ischemia by the dusky appearance of the kidneys, clamps were removed and reperfusion of the kidneys was achieved (as confirmed by the change of tissue color to pink). Muscle and skin layer were carefully closed, crocetin solution at a concentration of 50 mg/kg bodyweight was administered, intraperitoneal injection to the third group and appropriate analgesic (Ketamine 50 mg/kg bodyweight) were administered to all animals. Animals were sacrificed 24 h post-operation. Peripheral blood samples were collected at the time of experiment termination and kidneys were removed for further analysis.

Quantification of renal dysfunction based on biochemical parameters

To assess renal dysfunction in our experimental set up, blood samples were collected 24 h post-IRI from the right common carotid artery before animals were sacrificed. Serum samples were separated by centrifugation and analyzed by an automated biochemical analyzer (Olympus 600) for the levels of parameters currently in use for the assessment and monitoring of renal function, such as urea, creatinine, uric acid, and ions of Na, K, Cl, P, Mg and Ca.

Histological observation of renal injury

Both excised kidneys of each animal were formalin fixed and paraffin embedded. Sequentially, 5-mm-thick kidney sections were routinely stained with hematoxylin/eosin and carefully observed under light microscope to assess signs of renal tissue damage.

Detection of miRNA expression

RNA of small molecular weight was extracted from kidneys, using the mirVana miRNA isolation Kit (Ambion, Life Technologies, Waltham, MA). The RNA samples were reverse-transcribed to cDNA using the Applied Biosystems® TaqMan® MicroRNA Reverse Transcription Kit.

Real-time PCR was carried out using the TaqMan probes and the TaqMan Universal PCR Master Mix (Applied Biosystems, Foster City, CA), following the manufacturer’s instructions. The miR-132, miR-21 and miR-127 levels were measured and the input was normalized to U6-RNA. Data are represented as arbitrary units calculated with the ΔΔCt method and using as reference the levels of expression in heart.

Statistical analysis

All data are expressed as the mean (SD) of five animals per group. Differences among groups were analyzed by one-way analysis of variance with Graph Pad Prism for Windows and statistical analysis was performed using Student-Newman-Keuls test for comparison of two groups. The p < 0.05% was considered to be statistically significant.

## Results

Effects of crocetin in biochemical parameters

**Figure 1:**
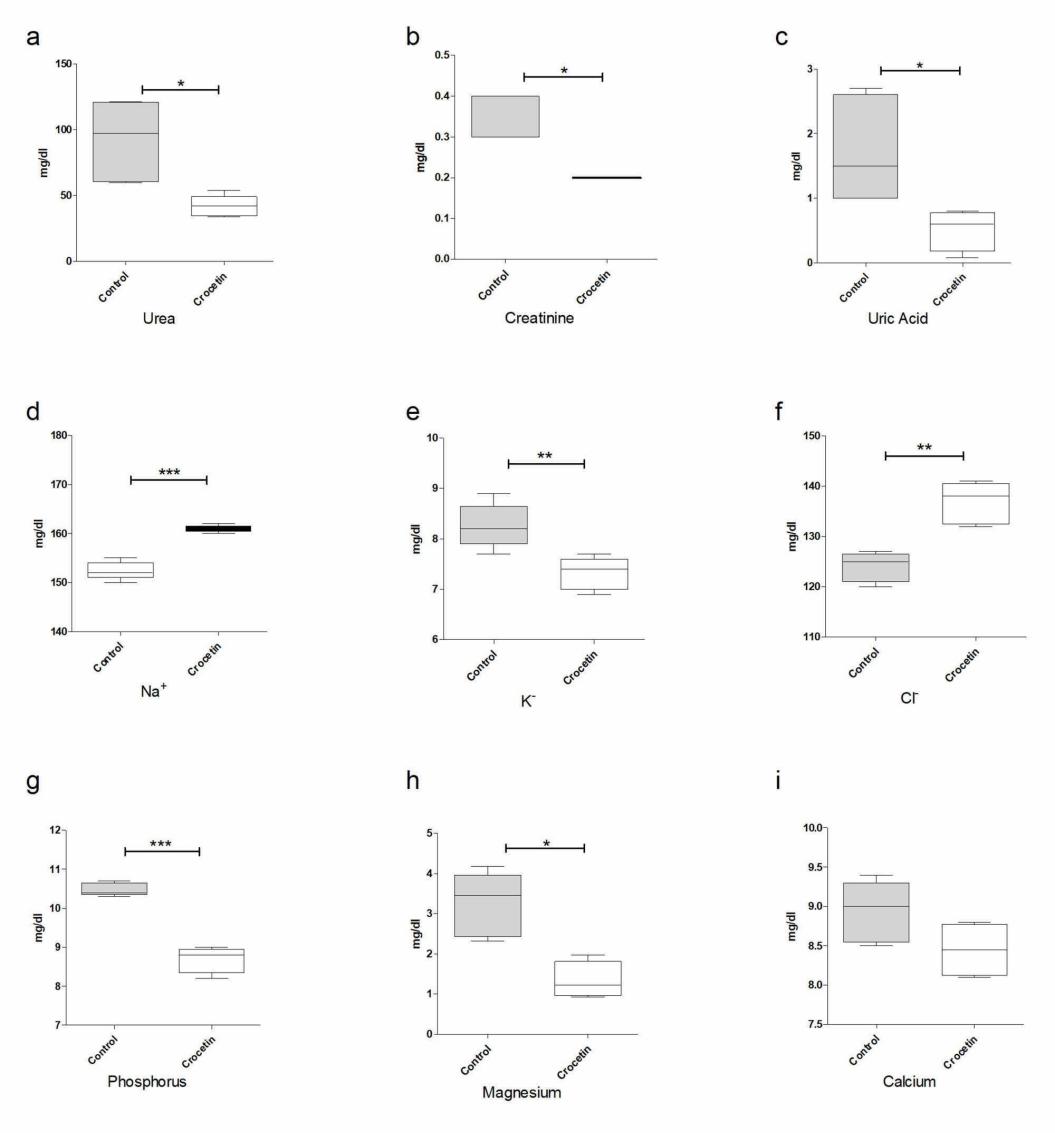
Effects of crocetin in biochemical parameters: (a) urea, (b) creatinine, (c) uric acid, (d) Na+, (e) K+, (f) Cl-, (g) phosphorus, (h) magnesium and (i) calcium

Figure 1 depicts the biochemical parameters determined in the peripheral blood of experimental animals 24 hours after artificial ischemia/reperfusion (I/R) and the administration of crocetin. The measured values ​​actually reflect the poor functioning of the kidneys. It is evident that the administration of crocetin, despite the short presence of the substance in the body, significantly affects these values ​​and causes them to decrease. More specifically, the statistical analysis showed the p-value to be statistically significant (<0.05%) in biochemical parameters analyzed (Figure 1).

Effects of crocetin in the histological entity of kidney

**Figure 2:**
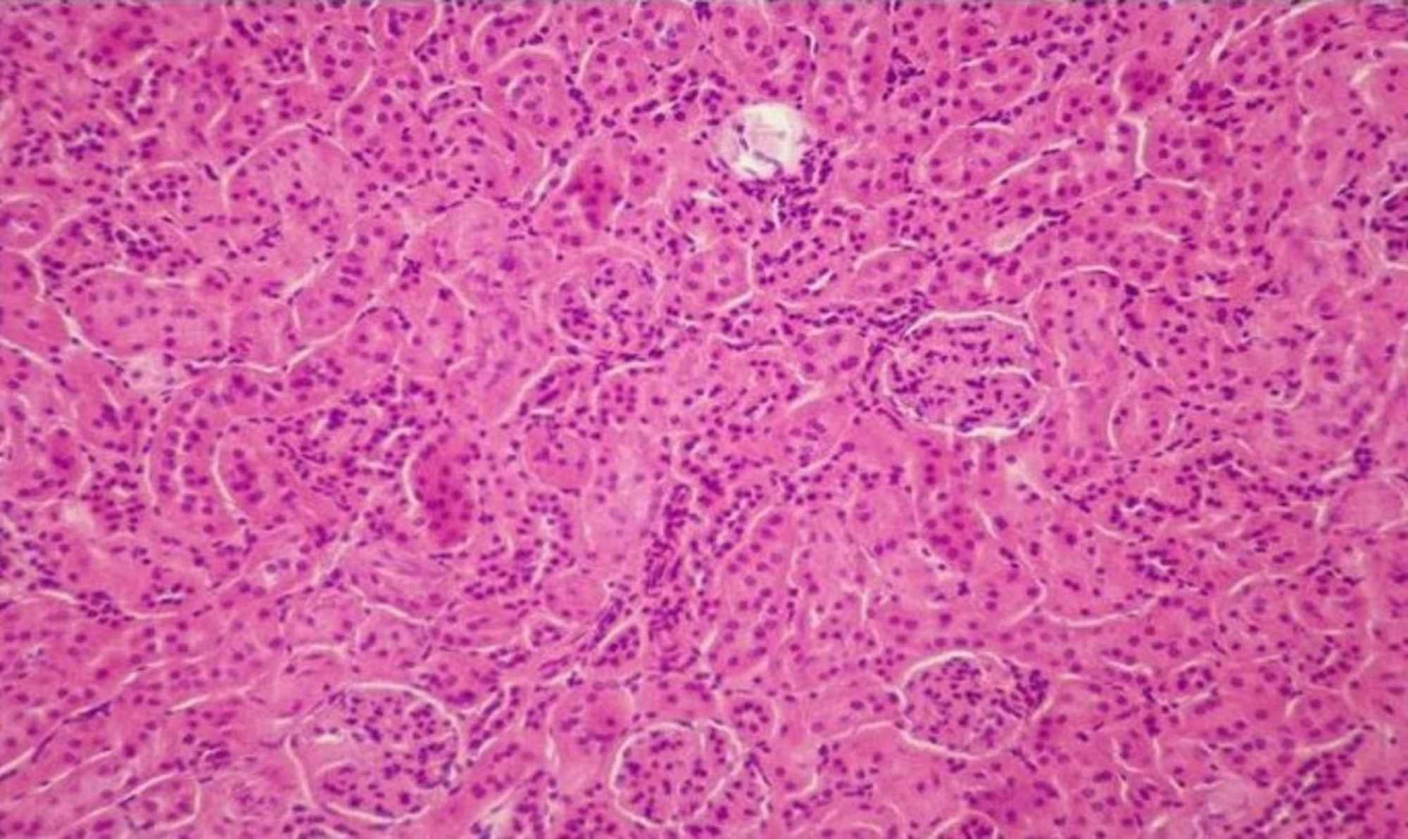
Non-injured tissue (H&E staining, 100x).

**Figure 3:**
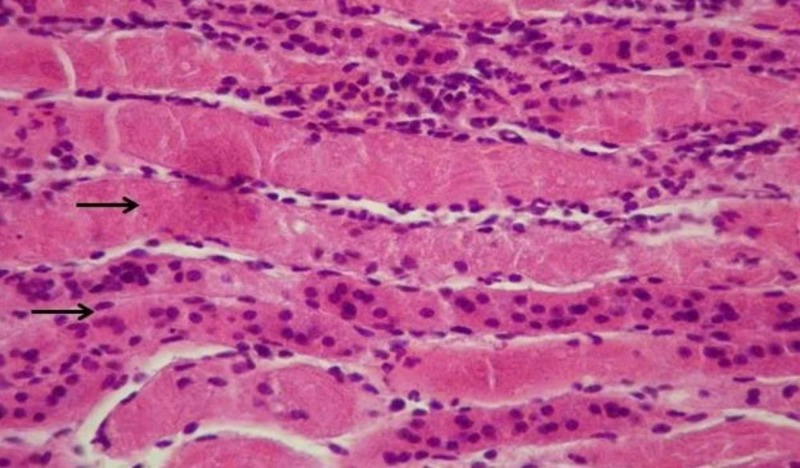
Ischemia reperfusion injury (IRI) – no crocetin (H&E staining, 200x). Arrows indicate shred epithelium and necrotic cells.

**Figure 4:**
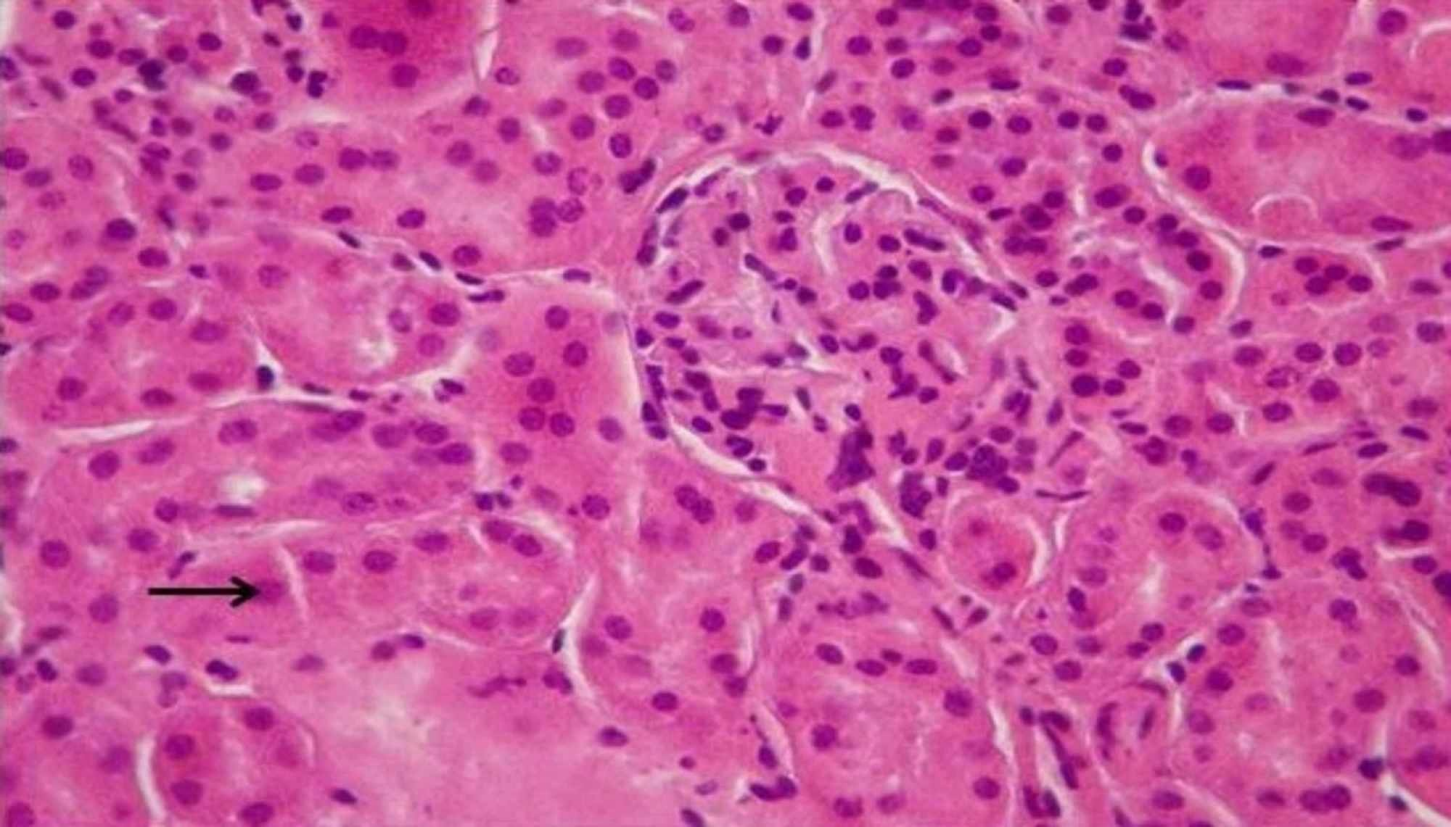
Ischemia reperfusion injury (IRI) with crocetin (H&E staining, 200x). Arrow indicates areas with less necrotic cells and no tissue damage.

**Figure 5:**
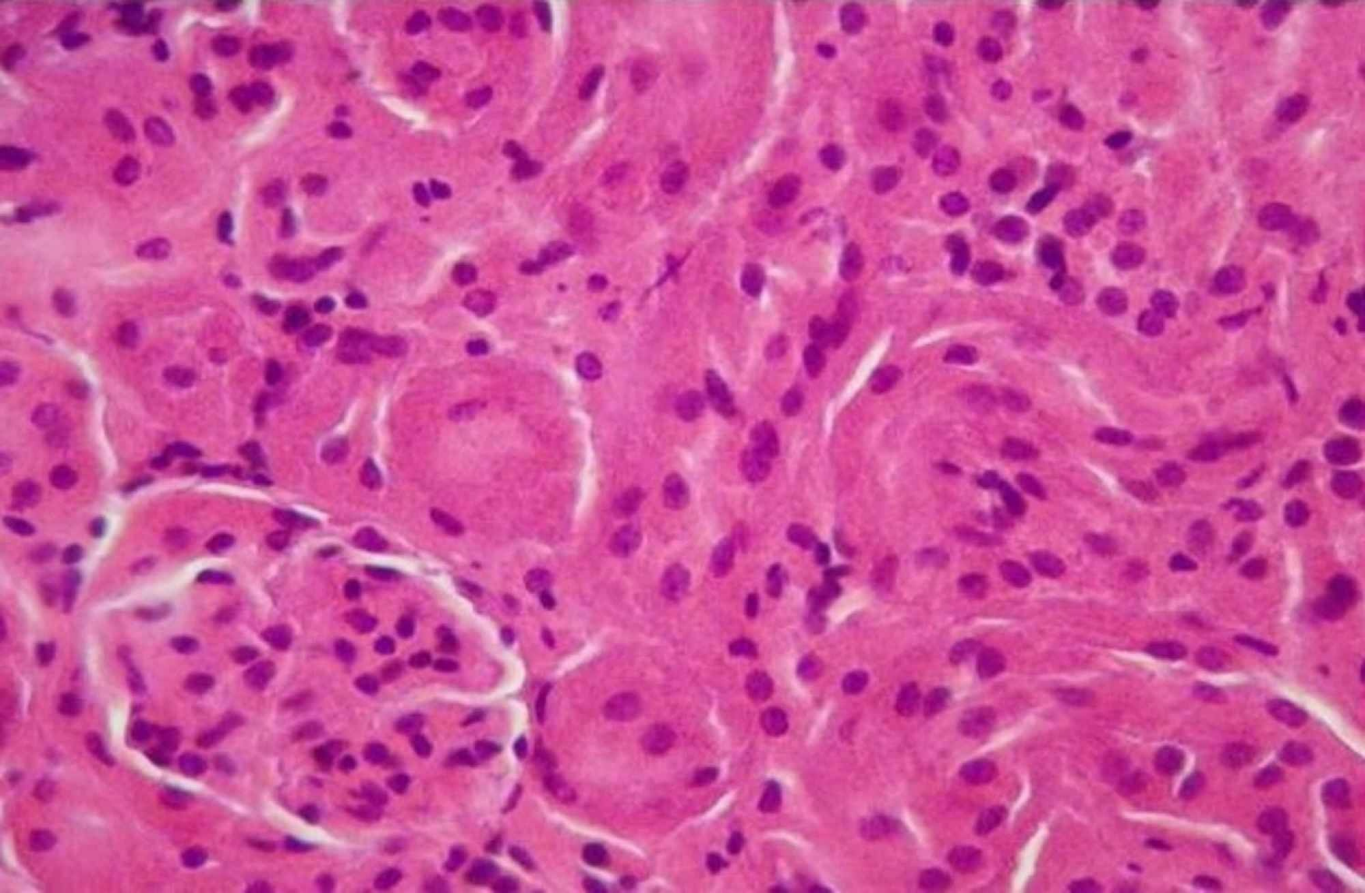
Ischemia reperfusion injury (IRI) and crocetin (H&E staining, 200x).

During microscopic observation of the incisions of the kidneys, lesions representative of the ischemic trauma were found. In particular, serious damages of the pyramidal structure, focal lumen dilation and signs of inflammation are observed. In addition, hyper-cellularity, increased interstitial fibrosis and damage to the limb capillaries were observed. These lesions are due to the increased number of necrotizing/apoptotic cells, forming the characteristic structure of the “shred” epithelium due to ischemic shock. These generalized lesions are mainly observed in the tissues of the IRI-group (Figures 2-5). However, it is evident in the crocetin group that the extent of the inflammation elements and the degree of epithelial damage is obviously limited (i.e., the extent of the “shred” epithelium).

Effects of crocetin administration in the expression profile miRNAs

**Figure 6:**
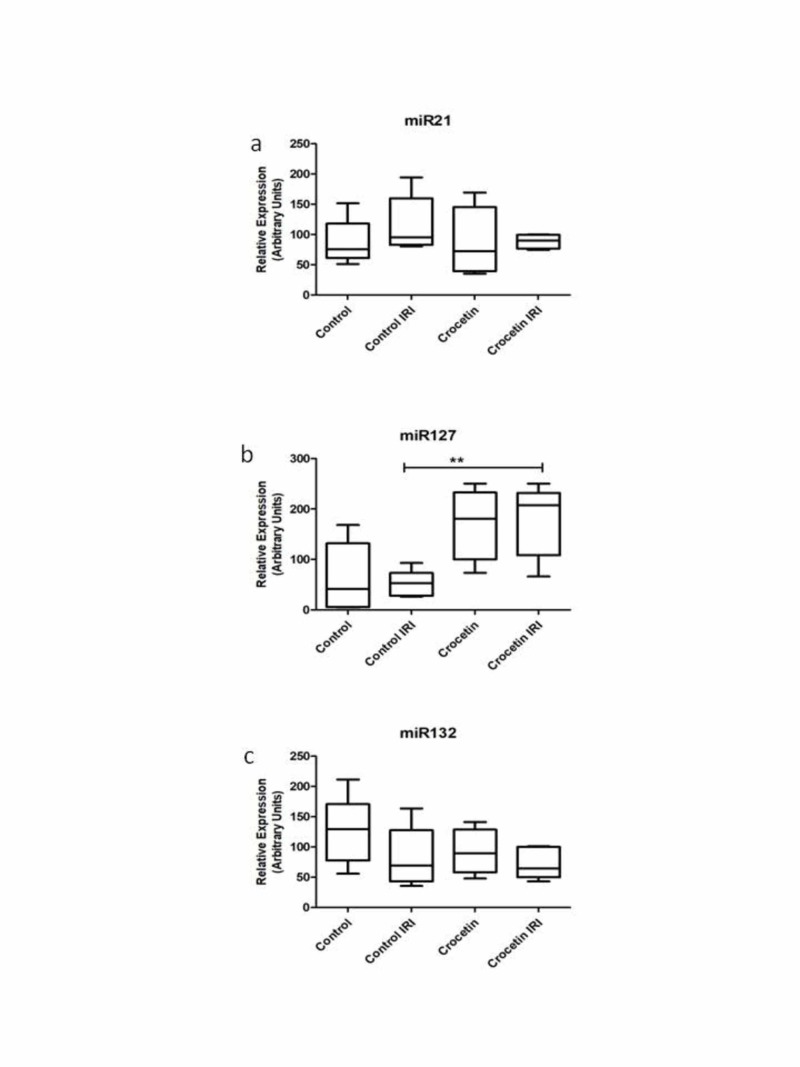
Effects of crocetin administration in the expression profile miRNAs (all four groups). IRI: Ischemia reperfusion injury; miRNA or miR: microRNA Effects of crocetin administration in the expression profile of microRNA-21 (panel a), microRNA-127 (panel b) and microRNA-132 (panel c). Bars represent arbitrary units of expression of each micro-RNA, as measured in each group of the study. Groups of the study: Control, Control IRI = animals with ischemia – reperfusion – injury, Crocetin = animals treated with crocetin without ischemia – reperfusion – injury and Crocetin-IRI group = animals with ischemia – reperfusion – injury and treated with crocetin.

Relative levels of expression of the three studied miRNAs in the kidneys are presented in Figure 6 as measured by RT-PCR. Specifically, miR21, miR127 and miR132 were chosen to be analyzed based on their known functions. It appears that levels of both miR21 and miR127 are elevated in the kidney tissues of untreated animals. The administration of crocetin appears to restore levels of expression.

## Discussion

The primary pathophysiological event occurring at the cellular level during ischemic damage is the dramatic drop in ATP levels. This causes a series of morphological, biochemical and physiological rearrangements. Increased production of free radicals of oxygen is an important mechanism by which damage to macromolecules, including membrane lipids, proteins and nucleic acids, is caused. A series of enzymes (such as catalases, dismutase peroxidase and glutathione peroxidase) as well as other molecules (such as tocopherols, vitamin C, glutathione, etc.) have been shown to offer little protection to the cell [8]. Based on this finding, modern efforts by scientists have turned to the search for natural products as alternative therapies to limit free radicals in the I/R injured kidney tissue.

In this context, the main objective of this study was to investigate the ability of crocetin to function as an alternative therapeutic molecule to treat renal failure due to I/R. In the literature, there are plenty of references to the strong antioxidant action of saffron and crocetin - one of its three key ingredients. The antioxidant activity of crocetin has been investigated both in relation to chemical toxicity and in pathological conditions from diseases such as cancer or degenerative, such as Alzheimer's and macular degeneration. Based on these studies, this paper used the ischemia/reperfusion model in mice and proceeded with the administration of crocetin once and further examining parameters to enhance our knowledge of this protective role.

Indeed, the results presented in the previous progress show that the administration of crocetin significantly changed the classical biochemical parameters used clinically to monitor renal function, such as urea, creatinine and uric acid levels in the blood. These findings are in full agreement with the bibliographic data.

In addition, we found that the presence of crocetin was able to greatly inhibit epithelial destruction and limit the number of dead/apoptotic cells. Obviously, this reversal of the histological image is due to the antioxidant action of crocetin that has protected a large number of ischemia/reperfusion epithelial cells from necrosis/apoptosis.

Of particular interest are the results of analysis of the expression profile of miRNAs. We refer to a family of micromolar RNAs that are not coded but are involved in post-transcriptional regulation. In recent years, studies have been continuously increasing, indicating how important the role of these small molecules is in regulating all cellular functions [14]. Indeed, a multitude of studies correlate the levels of expression of a miRNA with a specific pathological condition. Among these, studies report the altered expression of miR-21, miR-127 and miR-132, which were chosen to be explored in our model [15-17].

Of these, the miR-127 was the most interesting. In particular, levels of miR-127, showed a significant increase in the kidney tissue that had been treated with the I/R group of the crocetin-treated group. A recent study reports that this miRNA promotes the formation of focal adhesion complexes and enhances the interconnections between epithelial cells. Consequently, endocytosis is prevented, which acts prophylactically for epithelial cells [18].

## Conclusions

This article presents results that support the protective role of crocetin in the renal damage caused by ischemia/reperfusion shock. In addition, it appears that this action involves the induction of miR-127 expression. It would be of particular interest to investigate the potential for prophylactic action of this substance (i.e., the administration of crocetin prior to the induction of I/R shock) and its repeated administration after the shock to determine to what extent this substance is capable of inhibits the lesions caused. However, these proposals can form the basis for future work to demonstrate that miRNAs have the potential to be effective biomarkers and therapeutic targets for subsequent treatment. Therefore, miRNAs represent potential new therapeutic targets for a debilitating disease that continues to increase in prevalence worldwide and for which fully effective therapies are lacking.
